# The Spatial‐Temporal Dimension of Oncological Prevalence and Mortality in Romania

**DOI:** 10.1029/2023GH000901

**Published:** 2023-10-04

**Authors:** D. Peptenatu, I. D. Nedelcu, C. S. Pop, A. G. Simion, F. Furtunescu, M. Burcea, I. Andronache, M. Radulovic, H. F. Jelinek, H. Ahammer, A. K. Gruia, A. Grecu, M. C. Popa, V. Militaru, C. C. Drăghici, R. D. Pintilii

**Affiliations:** ^1^ Research Center for Integrated Analysis and Territorial Management—CAIMT Faculty of Geography University of Bucharest Bucharest Romania; ^2^ Carol Davila University of Medicine and Pharmacy Bucharest Romania; ^3^ Faculty of Administration and Business University of Bucharest Bucharest Romania; ^4^ Department of Experimental Oncology Institute of Oncology and Radiology of Serbia Belgrade Serbia; ^5^ Department of Biomedical Engineering and Healthcare Engineering Innovation Center Khalifa University Abu Dhabi United Arab Emirates; ^6^ Division of Medical Physics and Biophysics GSRC Medical University of Graz Graz Austria; ^7^ Faculty of Medicine Iuliu Haţieganu University of Medicine and Pharmacy Cluj‐Napoca Cluj‐Napoca Romania

**Keywords:** oncological prevalence, oncological mortality, persistence, continuity of persistence

## Abstract

The objective of this study was to identify spatial disparities in the distribution of cancer hotspots within Romania. Additionally, the research aimed to track prevailing trends in cancer prevalence and mortality according to a cancer type. The study covered the timeframe between 2008 and 2017, examining all 3,181 territorial administrative units. The analysis of spatial distribution relied on two key parameters. The first parameter, persistence, measured the duration for which cancer prevalence exceeded the 75th percentile threshold. Cancer prevalence refers to the total number of individuals in a population who have been diagnosed with cancer at a specific time point, including both newly diagnosed cases (occurrence) and existing cases. The second parameter, the time continuity of persistence, calculated the consecutive months during which cancer prevalence consistently surpassed the 75th percentile threshold. Notably, persistence of elevated values was also evident in lowland regions, devoid of any discernible direct connection to environmental conditions. In conclusion, this work bears substantial relevance to regional health policies, by aiding in the formulation of prevention strategies, while also fostering a deeper comprehension of the socioeconomic and environmental factors contributing to cancer.

## Key Points

- This study reveals geographical disparities in the prevalence of all cancer types in Romania
- The analysis spans the period from 2008 to 2017
- It was observed that cancer prevalence increased in correlation with anthropogenic interventions in the region

## 1 Introduction

Nearly 70% of cancer‐related deaths are reported in low‐ and middle‐income countries (World Health Statistics, 2018). Consequently, numerous studies have focused on exploring the geographical distribution of cancer to understand its occurrence across different geographic conditions. These investigations have provided valuable information, leading to new hypotheses regarding cancer causality (Al‐Ahmadi & Al‐Zahrani, 2013; Bhatt et al., 2013; Fowkes et al., 2013; Glass et al., 1995; Gou et al., 2017; Hassarangsee et al., 2015; Kitron & Kazmierczak, 1997; Ng et al., 2013; Oh et al., 2018; Wang et al., 2016; Xu et al., 2016) and the formulation of targeted public policies to address regional disparities (Teutsch & Churchill, 2000). The World Health Organization aims to promote health including reduction in noncommunicable diseases by identifying the socio‐economic disparities between developing and developed countries with incidence and prevalence of different groups of cancer types to ensure appropriate support that is relevant, flexible and effective and translates into measurable impact under the Country Cooperation Strategy Plan 2020 (World Health Organization, 2020). This strategy is based on extensive research that highlights potential causes of cancer and aids in the development of targeted prevention and intervention strategies to reduce cancer burden in high‐risk areas. Further, more comprehensive environmental monitoring and sustainable policy initiatives to protect public health and mitigate the effects of potential carcinogens in specific geographical locations is required (Larsen et al., 2023; Lynch & Rebbeck, 2013; Ribeiro & Fecht, 2021). By identifying spatial disparities in cancer distribution, policymakers including the WHO and NIH can target resources and interventions more effectively to address the underlying causes such as implementing stricter regulations on industrial emissions, improving access to healthcare services, or promoting healthier lifestyle choices. By adopting evidence‐based policies, governments and public health authorities can make a substantial impact on reducing cancer rates (Henley et al., 2014; Mohebbi et al., 2011; National Cancer Institute, 2016; World Health Organization, 2014). Understanding the long‐term patterns of cancer incidence in specific regions can provide valuable insights into the underlying factors contributing to elevated cancer risks in those areas. Such research helps identify cancer hotspots and their persistency, raising awareness of potential environmental hazards, lifestyle behaviors, and socioeconomic disparities that may be influencing cancer rates. By elucidating the specific geographical locations with high cancer burden and investigating the reasons for their persistence, policymakers and public health authorities can better tailor prevention and control measures to address the unique challenges faced by affected communities, ultimately leading to improved health outcomes and reduced cancer incidence (Hartley et al., 2017; Henley et al., 2017).

Enhancing our understanding of regional cancer distribution has been facilitated by the use of Geographical Information Systems (GIS) technology, which processes spatialized data, providing territorial significance to vast statistical databases. GIS utilization has led to the development of geospatial methodologies that support the formulation of cancer‐related public policies tailored to specific cancer types (Hay et al., 2013). Furthermore, the efficiency of GIS has been enhanced through the introduction of approaches that centralize the growing volume of geographical cancer distribution data (Craciunescu et al., 1999; Cross, 1997; Ferlay et al., 2016; Gazit et al., 1997; Hesterberg et al., 2006; Houston et al., 2004; Jemal et al., 2011; Landini & Rippin, 1993; Lefebvre et al., 1995; Mainster, 1990; Pohlman et al., 1996; Secomb et al., 1993; Torre et al., 2015; Vaupel, 1990; Zhu et al., 2002). Studies focusing on the geographical distribution of representative indicators for concentrations of various cancer types have demonstrated notable results using geospatial autocorrelation (Byng et al., 1996; Glick, 1979; Mandal et al., 2009; Rosenberg et al., 1999; Struewing et al., 1997). The geospatial analysis of cancer has contributed to a better understanding of the disease's determinants, with GIS providing the necessary tools to establish connections between territorial specificities, environmental conditions, and disease patterns (Correa, 1985; Correa et al., 2006; Crew & Neugut, 2006; Jarup et al., 2002; Onicescu et al., 2017; Zhang & Lawson, 2011). Collectively, geospatial correlation has the potential to become an indispensable tool for the tool for the planning and effectiveness of public policies in cancer diagnosis, and treatment (Allemani et al., 2015; Ferlay et al., 2015; Giacaman et al., 2009; Jones et al., 2006; Mosavi‐Jarrahi et al., 2007; Rainey et al., 2007; Rastogi et al., 2004; Siegel et al., 2016; Valsecchi & Steliarova‐Foucher, 2008). However, research has revealed that the lack of detailed data is a major limitation in identifying disparities in the distribution of various cancer types with respect to territorial differences. The aim of our current research is to utilize the Romanian cancer prevalence database at the territorial administrative unit (TAUs), which has been confirmed at the level of local institutions that collect data level for a detailed investigation of spatial and temporal determinants of cancer occurrence.

## 2 Materials and Methods

### 2.1 Study Area

**Figure 1:**
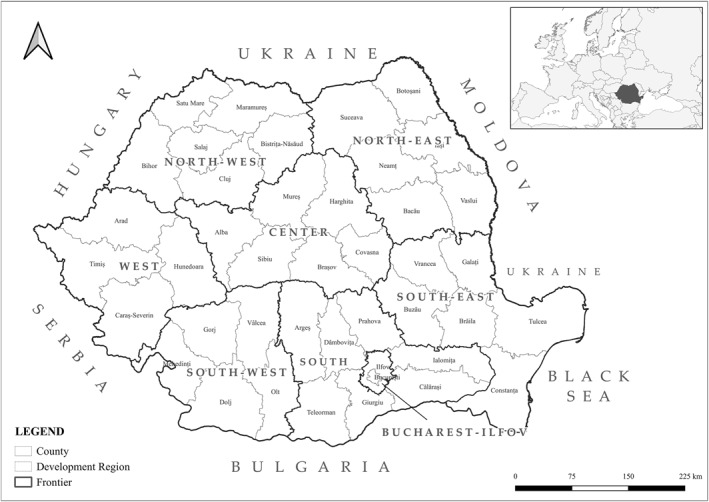
The territorial administrative structure of Romania.

The data analysis was conducted at the national level, encompassing all administrative units in Romania (Figure 1), and it spanned the period from 2008, when Romania became a member of the European Union, to 2017. Since 2008, Romania has adopted the reporting method recommended by the European Network of Cancer Registries, as specified in Romanian Order 2.027 issued on 26 November 2007.

### 2.2 Statistical Analysis

For the period 2008–2017, a database was gathered at the level of 3,181 TAU in Romania for all cancer codes, showing the number of yearly registered cancer cases and cancer mortality, collected according to the International Classification of Diseases Revision 10, which is currently used in the Romanian health system (Table 1). The data was obtained from the National School of Public Health, Management and Health Professional Development (http://www.snspms.ro/), public data. The data is accessed after a request is made.

**Table 1:** Malignancy Codes and Levels

Malignancy			
Code	Level I	Level II	Level III
C00–C14		**Malignancies, declared or presumed to be primary, with specified locations except those of lymphoid, hematopoietic and related tissue**	**Malignant tumors of the lip, oral cavity and pharynx**
C15–C26			**Primary malignancies found in the digestive organs**
C27–C29			
C30–C39			**Malignant tumors of the respiratory and intrathoracic organs**
C40–C41			**Malignant tumors of the bones and articular cartilages**
C42			
C43–C44			**Melanoma and other malignant tumors of the skin**
C45–C49			**Malignant tumors of mesothelial and soft tissues**
C50			**Malignant tumors of the breast**
C51–C58			**Malignant tumors of the female genitals**
C59			
C60–C63			**Malignant tumors of the male genitals**
C64–C68			**Malignant tumors of the urinary tract**
C69–C72			**Malignant tumors of the eye, brain and other parts of the central nervous system**
C73–C75			**Malignant tumors of the thyroid and other endocrine glands**
C76–C80		**Malignant tumors with poorly defined, secondary and unspecified areas**	

Level I encompassed malignancies categorized as C00–C96; Level II included primary malignancies with specified locations, excluding lymphoid, hematopoietic, and related tissues (C00–C75), as well as malignant tumors with poorly defined, secondary, and unspecified areas (C76–C80) and malignant tumors of lymphoid, hematopoietic, and related tissues (C81–C96); Level III consisted of specific tumor types, including malignant tumors of the lip, oral cavity, and pharynx (C00–C14), primary malignancies located in the digestive organs (C15–C26), malignant tumors of the respiratory and intrathoracic organs (C30–C39), malignant tumors of the bones and articular cartilages (C40–C41), melanoma and other malignant tumors of the skin (C43–C44), malignant tumors of mesothelial and soft tissues (C45–C49), malignant tumors of the breast (C50), malignant tumors of the female genitals (C51–C58), malignant tumors of the male genitals (C60–C63), malignant tumors of the urinary tract (C64–C68), and malignant tumors of the eye, brain, and other parts of the central nervous system (C69–C72), as well as malignant tumors of the thyroid and other endocrine glands (C73–C75).

For level, I, the total prevalence Pt, female Pf, and male Pm, expressed in absolute values, was analyzed. The total oncological fatality Dt, female Df, and male Dm was expressed in 1,000 people diagnosed with cancer

$$\text{Dt}=\frac{\text{Td}}{\text{Pt}}\times 1,000$$

where Dt refers to total cancer mortality, Td to total diagnosed and deceased persons, and Pt to total prevalence.

$$\text{Df}=\frac{\text{Tf}}{\text{Pf}}\times 1,000$$

Df refers to female cancer mortality, Tf to total female, diagnosed and deceased and Pf to female prevalence.

$$\text{Dm}=\frac{\text{Tm}}{\text{Pm}}\times 1,000$$

Dm refers to male cancer mortality, Tm to total males diagnosed and deceased and Pt to male prevalence.

For level II, the dynamics of prevalence for the period 2008–2017 was analyzed for the total values, both male and female, expressed in absolute values, as well as the average age of deaths for the total number of people with cancer—Vad t, as well as by gender (female‐Vad f and male‐Vad m). The analysis was done for all three categories of tumors: C00–C75, C76–C80, and C81–C96.

$$\text{Vad}\;t=\frac{V1+V2+V3+\text{⋯}+Vn}{n}$$

Vad t is the average age of deaths (total), *V*1 + *V*2 + *V*3 + ⋯ + *V*n—the age of death for each patient diagnosed and *n* being the number of patients (total).

$$\text{Vad}\;f=\frac{V1+V2+V3+\text{⋯}+Vn}{n}$$

Vad f is the average age of deaths (female), *V*1 + *V*2 + *V*3 + ⋯ + *V*n—the age of death for each patient diagnosed and *n* being the number of patients (female).

$$\text{Vad}\;m=\frac{V1+V2+V3+\text{⋯}+Vn}{n}$$

Vad m is the average age of deaths (male), *V*1 + *V*2 + *V*3 + ⋯ + *V*n—the age of death for each patient diagnosed and *n* being the number of patients (male).

Also, the average age of deaths for the types of tumors in the most widespread category in Romania, C00–C75, was analyzed. The mean age of death was analyzed for the following types: C00–C14, C15–C26, C30–C39, C40–C41, C43–C44, C45–C49, C50, C51–C58, C60–C63, C64–C68, C69–C72, C73–C75.

### 2.3 Spatial Analysis of Oncological Prevalence and Mortality

The total prevalence of females and males C00–C96 was calculated based on the processed medical database.

$$P=\frac{A}{B}\times 1,000$$

where *P* is prevalence, *A* is all new and pre‐existing cases during a given time and *B* is the population during the same time. The processing consisted in aggregating all cancer cases at the level of all TAU in Romania, to be processed later with a G.I.S. platform, used for cartographic modeling of statistical data. The resulting data tables were exported in CSV format and cartographic representations were made in the open‐source software, QuantumGIS 3.12.1, one of the most versatile tools for building spatial models.

### 2.4 Analysis of the Persistence and Continuity of Persistence of Cancer in a Geographical Area

Persistence is determined by calculating the total number of years in which the individual prevalence values for total, male and female cancer cases (coded as C00–C96) remained consistently higher than the threshold set at the 75th percentile. Continuity of persistence, in this context, refers to the longest duration throughout which instances of cancer cases maintain an uninterrupted state of prevalence surpassing the 75th percentile threshold for the specific codes C00–C96.

In order to assess both the extent of persistence and the continuous nature of persistence, we divided all geographical regions with recorded instances of cancer cases into four equal groups known as quartiles (Q1–4). In order to address values surpassing the 75th percentile threshold, which signify the highest prevalence, we utilized GIS technology. This allowed us to create three distinct sets of 10 binary images, customized for different groups: one set for the total population, another for females, and a third for males. Additionally, we generated 3,181 masks to align with each TAU. sets of 10 binary images corresponded to the total prevalence of cancer cases, as well as those specific to males and females (coded as C00–C96). In these images, areas surpassing the 75th percentile were represented by white, while those falling below were depicted in black. Our analysis of these images was conducted using the open‐source software ImageJ 1.53 (Schneider et al., 2012).

By employing the Subtract Tool within the ImageJ software, we successfully generated a comprehensive ensemble of 3,181 new image stacks, each meticulously tailored to correspond with individual years within the temporal span of 2008–2017. This construction process was orchestrated by extracting data from spatial masks delineating the geographic confines of TAU.

These stacks were crafted through an extraction process that involved the utilization of spatial masks outlining the boundaries of individual TAU. This extraction was carried out utilizing the data contained within the three distinct sets of 10 binary images. This process ultimately led to the creation of a single image for each year, capturing the conditions during that specific period.

Within each of the 3,181 new sets, there were 10 images corresponding to the years between 2008 and 2017. If, in a particular year, the TAU being analyzed exhibited a prevalence surpassing the 75th percentile threshold, the corresponding image depicted the TAU as white. Conversely, if the prevalence fell below the 75th percentile, the image for that year remained entirely black.

This approach played a crucial role in depicting intricate prevalence patterns not only for the total population but also for male, female, and overall cancer cases that exceed the rigorous 75th percentile threshold. Such detailed analysis significantly enhances our understanding within the realm of medical geography.

For the persistence, we established three empirically defined categories: low persistence (0–3 years), medium persistence (4–6 years), and high persistence (7–10 years). These categories were assigned visual representations using color coding facilitated by the Image‐Color‐Channel Tools. Specifically, gray color was assigned to denote persistence of less than 3 years, green represented 4–6 years, and red indicated 7–10 years of persistence.

Following this, we utilized the Image‐Stack‐Z Project function to convert these three persistence categories into three distinct color‐coded images (gray, green and red). By leveraging the Process‐Image Calculator‐Add feature, we overlaid the color images from the prior step and generated of comprehensive persistence maps. These maps effectively depicted the prevalence of total, male, and female cancer instances coded as C00–C96.

Regarding the continuity of persistence, each TAU was classified into one of three categories. This categorization was carried out using the Image‐Color‐Channel Tools, where we assigned color codes to the three stacks in the following manner: gray indicated an unbroken continuity of persistence lasting 0–5 years, red denoted 6–9 years, and blue represented 10 continuous years.

Subsequently, through the utilization of the Image‐Stack‐Z Project function, we converted the three stacks into three distinct images. Finally, by utilizing the Process‐Image Calculator‐Add function, we merged the resultant three‐color images. This comprehensive amalgamation yielded a map that effectively illustrated the unbroken continuity of persistence.

In terms of persistence and continuity of persistence, our detailed analysis of cancer prevalence patterns revealed three distinct classes of persistence and outlined how these have evolved over the years. These results contribute significantly to our understanding of changes in cancer prevalence in territorial administrative contexts.

## 3 Results

### 3.1 General Characteristics About the Prevalence and Fatality of Cancer in Romania

Table 2 shows the time‐course of the number of cancer cases diagnosed between 2008 and 2017 for total values (Pt), and distributed by gender (Pf, Pm), for malignant tumors C00–C96 and for categories C00–C75, C76–C80, and C81–C96. The analysis of geographical distribution shows the predominance of C00–C75, with 77.9% of the malignant tumors registered in Romania in 2008%, and 78.3% at the end of the analysis period.

**Table 2:** Prevalence of Malignant Tumors in Romania Between 2008 and 2017

Cases	2008	2009	2010	2011	2012	2013	2014	2015	2016	2017
Pt C00–C96	320,346	329,118	325,624	314,752	302,438	309,699	304,364	283,869	276,560	271,799
Pf C00–C96	157,524	159,701	156,600	149,920	143,773	148,017	145,083	132,649	129,078	127,128
Pm C00–C96	162,822	169,417	169,024	164,832	158,665	161,682	159,281	151,220	147,482	144,671
Pt C00–C75	249,423	253,425	253,471	241,897	232,342	236,307	232,837	220,947	217,032	212,720
Pf C00–C75	122,256	122,289	121,376	113,360	108,675	111,159	109,737	102,314	100,479	98,666
Pm C00–C75	127,167	131,136	132,095	128,537	123,667	125,148	123,100	118,633	116,553	114,054
C00–C75/C00–C96%	77.9	77.0	77.8	76.9	76.8	76.3	76.5	77.8	78.5	78.3
Pt C76–C80	24,002	28,177	23,863	25,796	24,466	26,055	26,948	23,555	21,708	21,545
Pf C76–C80	12,974	14,877	12,436	13,952	13,507	14,057	14,358	12,228	11,227	11,006
Pm C76–C80	11,028	13,300	11,427	11,844	10,959	11,998	12,590	11,327	10,481	10,539
C76–C80/C00–C96%	7.5	8.6	7.3	8.2	8.1	8.4	8.9	8.3	7.9	7.9
Pt C81–C96	46,921	47,516	48,290	47,059	45,630	47,337	44,579	39,367	37,820	37,534
Pf C81–C96	22,294	22,535	22,788	22,608	21,591	22,801	20,988	18,107	17,372	17,456
Pm C81–C96	24,627	24,981	25,502	24,451	24,039	24,536	23,591	21,260	20,448	20,078
C81–C96/C00–C96%	14.6	14.4	14.8	15.0	15.1	15.3	14.6	13.9	13.7	13.8

*Note.* Pt, total prevalence; Pf, female prevalence; Pm, male prevalence; C00–C75, Malignancies, declared or presumed to be primary, with specified locations except those of lymphoid, hematopoietic and related tissue; C76–C80, malignant tumors with poorly defined, secondary, and unspecified locations; C81–C96, malignant tumors of lymphoid, hematopoietic and related tissues.

The analysis of cancer fatality showed a sharp increase, from 60.30‰ in 2008 to 79.50‰ in 2017. Furthermore, the research by gender showed a pronounced increase in female cancer mortality, from 51.50‰, in 2008, to 72.10‰ in 2017, and male fatality from 69.10‰ in 2008 to 86.80‰ (Table 3).

**Table 3:** Cancer Fatality in Romania

	2008	2009	2010	2011	2012	2013	2014	2015	2016	2017
Dc t C00–C96‰	60.3	59.6	61	63.11	67.5	68.1	70	77	79.1	79.5
Dc f C00–C96‰	51.5	52.2	53.5	56.1	59.7	61.3	62	70.1	71.9	72.1
Dc m C00–C96‰	69.1	67.1	68.5	70.1	75.3	74.8	77.9	84	86.3	86.8

*Note.* Dc t, total cancer fatality; Dc f, female cancer fatality; Dc m, male cancer fatality, C00–C96, malignant tumors.

The analysis of the average age of oncological deaths, calculated for the three categories of malignant tumors, including the total number and determined by gender, shows the increase in age throughout the analyzed period. For the dominant tumor category C00–C75, the average age of oncological deaths increased from 60.5‰ in 2008 to 68.6‰ in 2017. The analysis by gender for the same categories of tumors showed an increase in the average age of oncological deaths for the total patients, while the increase was more pronounced in the female population (Table 4).

**Table 4:** The Average Age of Oncological Deaths in Romania

Average age of death	2008	2009	2010	2011	2012	2013	2014	2015	2016	2017
Vad t C00–C75	60.5	61.2	61.4	61.6	70.5	67	67.3	67.3	67.8	68.6
Vad f C00–C75	60.7	61.3	61.6	61.7	74.9	67	67.7	67.5	68.1	69.2
Vad m C00–C75	60.3	61.1	61.2	61.6	66.1	67	66.9	67.1	67.4	67.9
Vad t C76–C80	63.5	64.1	64.3	65.3	70.5	65.3	68.7	68.6	68.5	68.9
Vad f C76–C80	63.8	64.1	64.8	65.8	77	65.5	71.4	70.6	69.8	70.3
Vad m C76–C80	63.3	64.1	63.9	64.8	64.1	65	65.9	66.6	67.1	67.4
Vad t C81–C96	61.5	61.5	61.9	62.9	65.8	63.3	67.4	66.3	67.7	68.5
Vad f C81–C96	61.6	61.8	61.4	63	64.6	63.7	67.1	66.3	67.3	67.9
Vad m C81–C96	61.5	61.1	62.4	62.8	67	62.9	67.7	66.3	68.1	69.1

*Note.* Vad t, average age of deaths for the total number of people with cancer; Vad f, the average age of deaths for women with cancer; Vad m, the average age of deaths for people male cancer patients; C00–C75, malignancies, declared or suspected to be primary, with specified locations other than lymphoid, hematopoietic and related tissue; C76–C80, malignancies with poorly defined, secondary and unspecified areas; C81–C96, malignant tumors of lymphoid, hematopoietic and related tissues.

**Figure 2:**
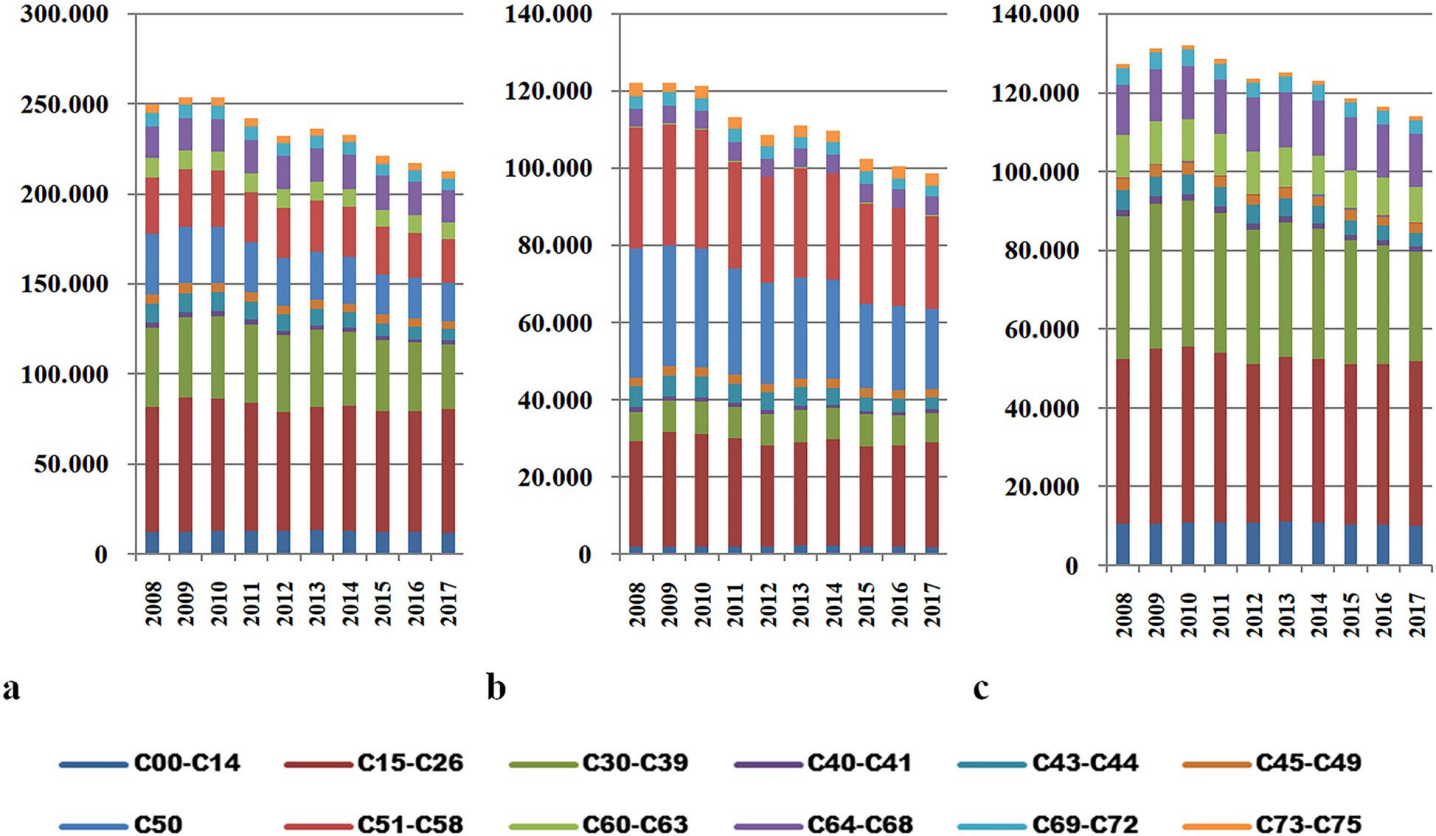
The geographical distribution of tumor types within the C00–75 range, displaying the number of diagnosed cases. The figure includes three subfigures: (a) total cases, (b) female cases, and (c) male cases. C00–C14, malignant tumors of the lip, oral cavity, and pharynx; C15–C26, primary malignancies located in the digestive organs; C30–C39, malignant tumors of the respiratory and intrathoracic organs; C40–C41, malignant tumors of the bones and articular cartilages; C43–C44, melanoma and other malignant tumors of the skin; C45–C49, malignant tumors of mesothelial and soft tissues; C50, malignant tumors of the breast; C51–C58, malignant tumors of female genitals; C60–C63, malignant tumors of male genitals; C64–C68, malignant tumors of the urinary tract; C69–C72, malignant tumors of the eye, brain and other parts of the central nervous system; C73–C75, malignant tumors of the thyroid and other endocrine glands. Data source: Ministry of Health.

Throughout the analyzed period, this category exhibited the highest number of cases. In terms of total values, primary malignancies located in the digestive organs (C15–C26) were the most widespread, with approximately 69,000 cases over the 10‐year period. They were followed by malignant tumors of the respiratory and intrathoracic organs (C30–C39), peaking at over 45,000 cases in 2010, and malignant tumors of the breast (C50), with the highest number of cases recorded in 2008, reaching approximately 34,000 new diagnoses (Figure 2).

In the female population, the prevalence was dominated by malignant breast tumors (C50) with 33,356 cases registered in 2008, declining to 20,748 in the last year of analysis. Malignant tumors of the female genitals (C51–C58) accounted for approximately 31,000 records in 2008 and 24,000 in 2017. Primary malignancies located in the digestive organs (C15–C26) registered 29,750 cases in 2009. Together, these three categories represented over 60% of the total C00–C75 cases in the female population.

Among males, primary malignancies in the digestive organs (C15–C26) took the lead with over 41,000 records, followed by malignant tumors of the respiratory and intrathoracic organs (C30–C39) with over 36,000 cases diagnosed over the 10‐year period.

To provide a more comprehensive overview of the distribution of dominant tumors in Romania, we have compiled total values for each tumor type within the C00–C96 range. Tables 3, 4, 5 display the proportions of each tumor type. The analysis reveals the following insights:

**Table 5:** Share of Tumor Types in the C00–C75 Category for Total Malignancies C00–C96 (%)

Year	C00–C14	C15–C26	C30–C39	C40–C41	C43–C44	C45–C49	C50	C51–C58	C60–C63	C64–C68	C69–C72	C73–C75
2008	3.8	21.6	13.7	0.9	3.3	1.6	10.5	9.9	3.3	5.5	2.3	1.4
2009	3.8	22.6	13.7	0.9	3.2	1.6	9.6	9.6	3.3	5.5	2.3	1.1
2010	3.9	22.6	14.0	0.8	3.2	1.6	9.6	9.5	3.3	5.6	2.3	1.3
2011	4.1	22.6	13.9	0.8	3.2	1.5	8.9	8.9	3.3	5.9	2.4	1.4
2012	4.2	22.0	14.0	0.8	3.1	1.6	8.7	9.2	3.6	6.1	2.2	1.4
2013	4.3	22.1	13.8	0.7	3.0	1.5	8.6	9.3	3.3	6.1	2.2	1.3
2014	4.2	22.7	13.6	0.7	2.8	1.6	8.5	9.0	3.3	6.2	2.3	1.4
2015	4.4	23.5	14.0	0.8	2.5	1.7	7.8	9.3	3.4	6.5	2.4	1.5
2016	4.4	24.3	13.7	0.8	2.5	1.6	8.0	9.2	3.5	6.7	2.3	1.5
2017	4.3	25.4	13.1	0.8	2.4	1.6	7.7	9.0	3.4	6.7	2.3	1.6

In terms of total malignant tumors, primary malignancies located in the digestive organs (C15–C26) hold significant weight, accounting for over 210 per 1,000 cases in the span of 10 years. Malignant tumors of the female genitals (C51–C58) show a decreasing share, declining from 9.90 per 1,000 cases to 9.00 per 1,000 cases by 2017. Conversely, malignant tumors of the respiratory and intrathoracic organs (C30–C39) demonstrate a share of 13.10 per 1,000 cases in 2017.

Furthermore, the geographical distribution analysis highlights an increasing percentage of primary malignancies in the digestive organs (C15–C26) and malignant tumors of the urinary tract (C64–C68). Their combined share among total tumors rises from 5.50 per 1,000 cases in 2008 to 6.70 per 1,000 cases in 2017.

Table 6 provides valuable insights into the dynamics of tumor types within category C00–C75 for the female population. Three specific tumor types of collectively account for over 50% of all malignancies in the C00–C96 range: malignant tumors of the breast (C50), malignant tumors of the female genitals (C51–C58), and primary malignancies in the digestive organs (C15–C26). Notably, there is a notable trend of increasing shares for these tumor types over the period analyzed.

**Table 6:** Share of Female C00–C75 Tumor Types in Total Malignant Tumors C00–C96—Female (%)

Year	C00–C14	C15–C26	C30–C39	C40–C41	C43–C44	C45–C49	C50	C51–C58	C69–C72	C73–C75
2008	1.1	17.4	4.9	0.8	3.4	1.5	21.2	20.1	2.1	2.2
2009	1.2	18.6	5.0	0.7	3.4	1.6	19.6	19.7	2.1	1.7
2010	1.2	18.6	5.4	0.7	3.4	1.6	19.7	19.8	2.1	2.0
2011	1.3	18.7	5.4	0.7	3.4	1.5	18.4	18.7	2.3	2.1
2012	1.3	18.3	5.7	0.6	3.2	1.6	18.1	19.2	2.1	2.1
2013	1.4	18.0	5.8	0.6	3.2	1.5	17.7	19.4	2.1	2.1
2014	1.5	19.0	5.6	0.6	3.0	1.6	17.7	19.0	2.1	2.1
2015	1.5	19.6	6.2	0.7	2.7	1.7	16.5	19.8	2.3	2.4
2016	1.4	20.4	6.1	0.6	2.7	1.7	16.9	19.6	2.2	2.4
2017	1.3	21.4	6.0	0.8	2.4	1.8	16.3	19.2	2.2	2.6

The share of primary malignancies found in the digestive organs (C15–C26) has shown an upward trajectory, rising from 17.400 per 1,000 cases in 2008 to 21.40 per 1,000 cases in 2017. Similarly, malignant tumors of the respiratory and intrathoracic organs (C30–C39) have witnessed an increase in their share, growing from 4.90 per 1,000 cases in 2008 to 6.00 per 1,000 cases in 2017. Malignant tumors of mesothelial and soft tissues (C45–C49) also experienced a slight rise, from 1.50 per 1,000 cases in 2008 to 1.80 per 1,000 cases in 2017.

Table 7 emphasizes the significant proportion of primary malignancies located in the digestive organs (C15–C26). This share has exhibited an upward trend throughout the analyzed period, rising from 25.80 per 1,000 cases in 2008 to 28.90 per 1,000 cases in 2017. Conversely, malignant tumors of the respiratory and intrathoracic organs (C30–C39) experienced a slight decrease, declining from 22.20 per 1,000 cases in 2008 to 19.30 per 1,000 cases in 2017.

**Table 7:** Share of Male C00–C75 in Total Male Malignancies C00–C96 (%)

Year	C00–C14	C15–C26	C30–C39	C40–C41	C43–C44	C45–C49	C50	C60–C63	C64–C68	C69–C72	C73–C75
2008	6.5	25.8	22.2	1.1	3.1	1.7	0.2	6.6	7.9	2.5	0.6
2009	6.2	26.2	21.8	1.1	3.0	1.7	0.2	6.3	7.9	2.5	0.6
2010	6.5	26.4	21.9	1.0	3.0	1.7	0.3	6.3	8.0	2.5	0.6
2011	6.6	26.2	21.6	1.0	3.1	1.5	0.2	6.3	8.4	2.5	0.7
2012	6.8	25.3	21.6	1.0	3.0	1.6	0.2	6.8	8.7	2.3	0.7
2013	6.9	25.9	21.2	0.9	2.8	1.6	0.2	6.3	8.7	2.4	0.6
2014	6.8	26.1	20.8	0.8	2.7	1.6	0.2	6.3	8.8	2.5	0.7
2015	6.9	26.9	20.8	0.9	2.4	1.8	0.2	6.4	9.0	2.4	0.7
2016	6.9	27.8	20.4	0.9	2.4	1.6	0.2	6.5	9.2	2.4	0.7
2017	6.9	28.9	19.3	0.8	2.4	1.6	0.2	6.4	9.2	2.5	0.7

**Figure 3:**
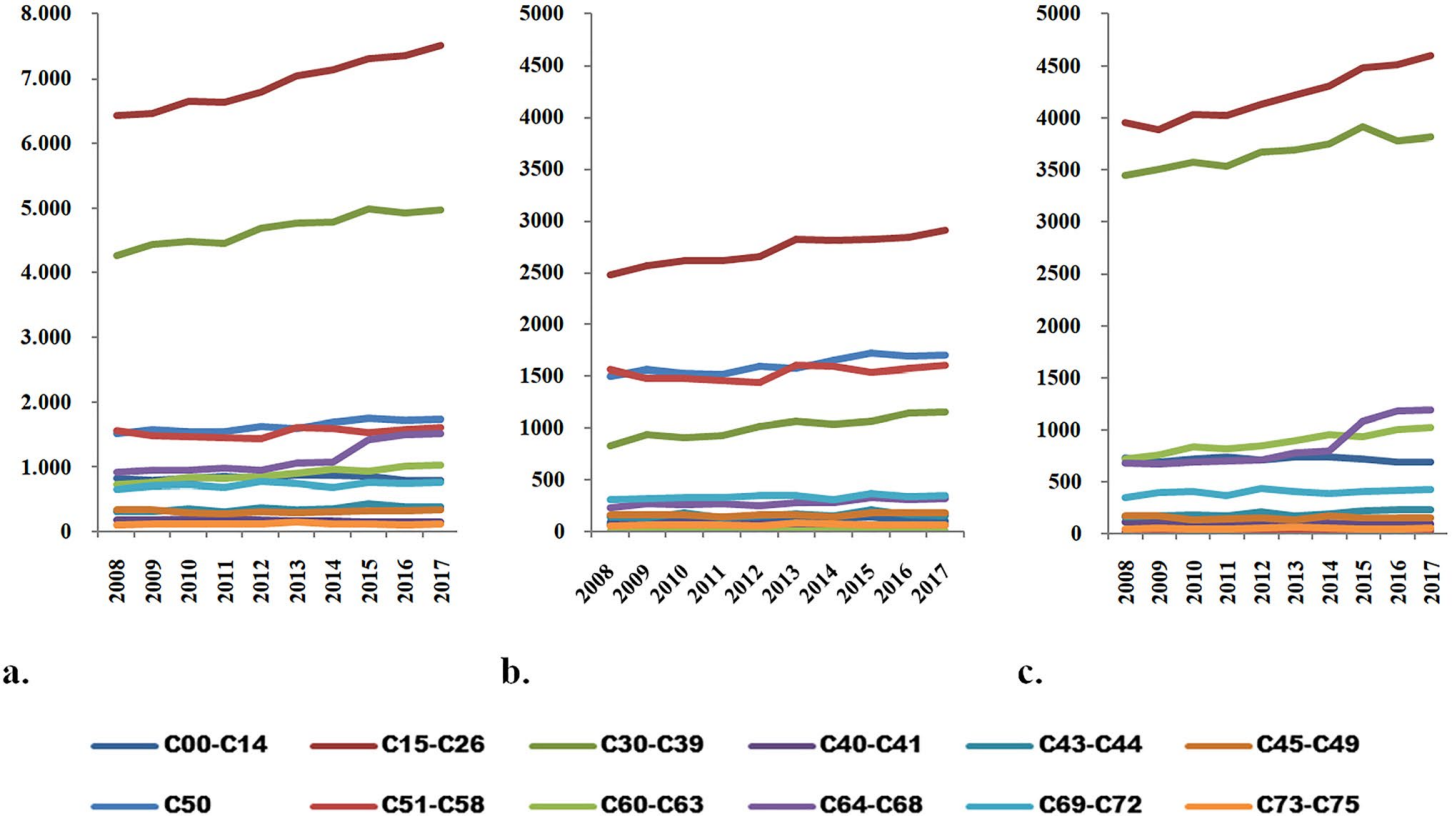
The temporal trends in death rates attributed to the leading causes, categorized as follows: (a) total deaths, (b) female deaths, and (c) male deaths. The causes of death are classified according to the following categories: C00–C14, malignant tumors of the lip, oral cavity, and pharynx; C15–C26, primary malignancies located in the digestive organs; C30–C39, malignant tumors of the respiratory and intrathoracic organs; C40–C41, malignant tumors of articular bones and cartilage; C43–C44, melanoma and other malignant tumors of the skin; C45–C49, malignant tumors of mesothelial and soft tissues; C50, malignant tumors of the breast; C51–C58, malignant tumors of female genitals; C60–C63, malignant tumors of male genitals; C64–C68, malignant tumors of the urinary tract; C69–C72, malignant tumors of the eye, brain, and other parts of the central nervous system; C73–C75, malignant tumors of the thyroid and other endocrine glands. The data source is the Ministry of Health.

Figure 3 depicts the number of deaths attributed to the most prevalent tumor types. Notably, primary malignancies located in the digestive organs (C15–C26) accounted for a substantial number of deaths, reaching approximately 7,500 in 2017, exhibiting a sharp increase over time. Similarly, malignant tumors of the respiratory and intrathoracic organs (C30–C39) contributed to a remarkably high number of deaths, with over 4,500 recorded annually, indicating a consistent upward trend throughout the period analyzed.

Female cancer fatality exhibited a distinctive profile, with the highest number of deaths attributed to metastases localized in the digestive organs (C15–C26), reaching approximately 2900 deaths in 2017. Additionally, breast cancer (C50) accounted for 1,700 deaths in 2017, while malignant tumors of the female genitals (C51–C58) resulted in around 1,600 deaths. Notably, all these malignancies demonstrated an increasing number of deaths each year.

In terms of male cancer fatality, a prominent feature was the significant number of deaths caused by metastases found in the digestive organs (C15–C26), with over 3,800 deaths annually. Additionally, deaths associated with respiratory and intrathoracic malignancies (C30–C39) exceeded 3,400 each year.

**Figure 4:**
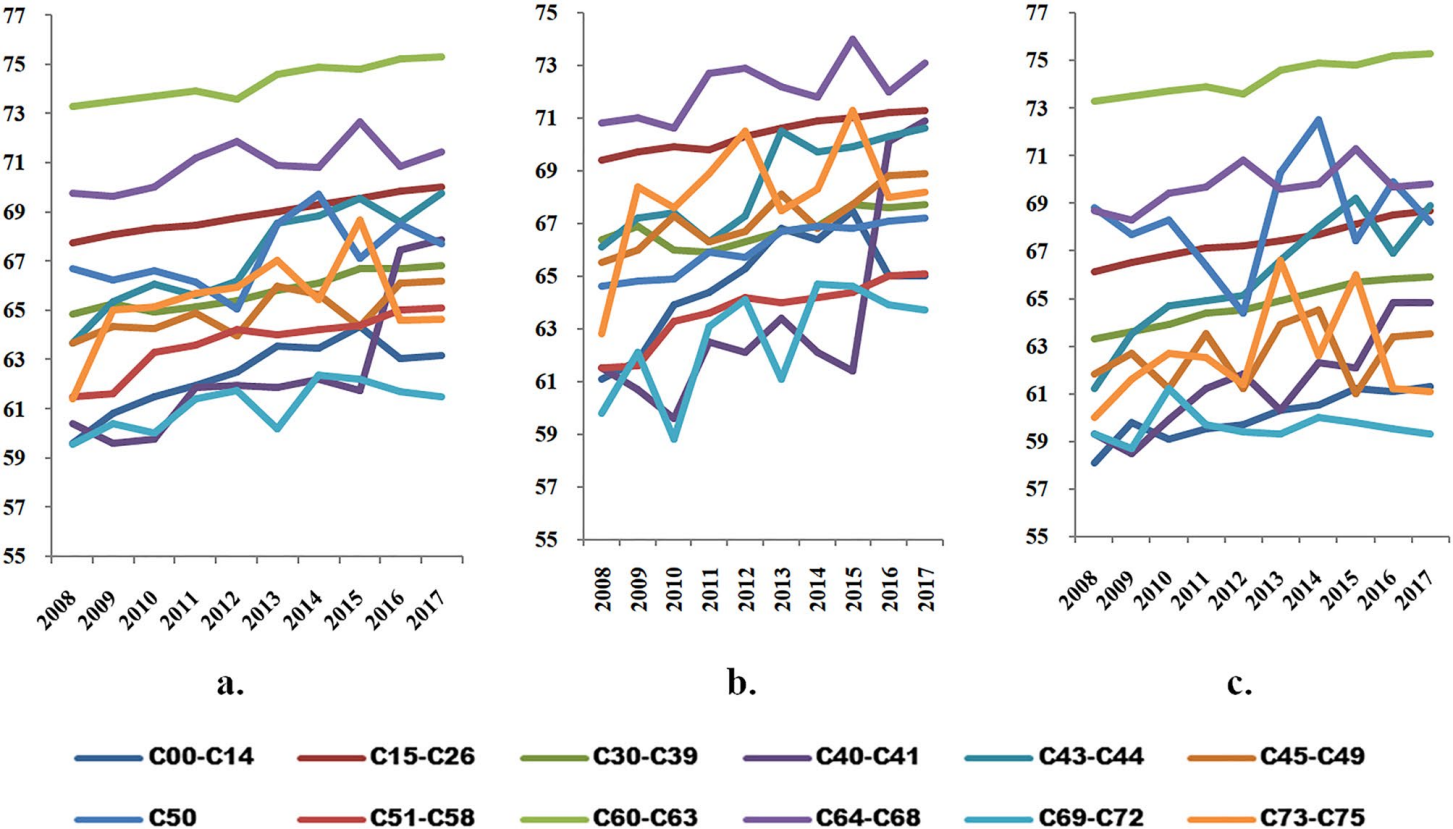
The temporal patterns of the average age at death for each specific type of tumor, categorized as follows: (a) total deaths, (b) female deaths, and (c) male deaths. The corresponding codes and tumor types are as follows: C00–C14, malignant tumors of the lip, oral cavity, and pharynx; C15–C26, primary malignancies located in the digestive organs; C30–C39, malignant tumors of the respiratory and intrathoracic organs; C40–C41, malignant tumors of articular bones and cartilage; C43–C44, melanoma and other malignant tumors of the skin; C45–C49, malignant tumors of mesothelial and soft tissues; C50, malignant tumors of the breast; C51–C58, malignant tumors of female genitals; C60–C63, malignant tumors of male genitals; C64–C68, malignant tumors of the urinary tract; C69–C72, malignant tumors of the eye, brain, and other parts of the central nervous system; C73–C75, malignant tumors of the thyroid and other endocrine glands. The Ministry of Health serves as the data source for this information.

Figure 4 presents the temporal patterns of the average age at death for each tumor type within the C00–C75 category. Notably, there was a general increase in the average age for most tumor types leading to death. However, malignant eye tumors (C69–C72) exhibited a contrary trend, with a decrease in average age over the analyzed period. Analyzing the data for all tumors combined revealed that the male population had significantly higher average ages for patients with malignant tumors of the male genitals (C60–C63), which increased from 73 to 75 years between 2008 and 2017. Similarly, patients with malignant tumors of the urinary tract (C64–C68) had an average age ranging from 69 to 71 years. In contrast, malignant eye tumors (C69–C72) consistently had low average ages, with a minimum of 59 years throughout the entire period.

Examining the same analysis for the female population revealed significant changes in the hierarchy of tumor types. Malignant tumors of the urinary tract (C64–C68) exhibited the highest prevalence, with an average age ranging from 70 to 73 years. Primary malignancies in the digestive organs (C15–C26) also showed high average ages, increasing from 69 to 71 years. Conversely, malignant eye tumors (C69–C72) had the lowest average ages, reaching 63 years in 2017. Notably, malignant tumors of bones and articular cartilage (C40–C41) were diagnosed in an aging population, with an average age reaching 71 in 2017.

In the male population, the average age at death was highest for malignant tumors of the male genitals (C60–C63), surpassing 73 years, and for malignant tumors of the urinary tract (C64–C68), exceeding 70 years. On the other hand, malignant tumors of the lip, oral cavity, and pharynx (C00–C14) had the lowest prevalence, with the average age at death increasing from 58 to 61 years. Malignant tumors of the eye (C69–C72) also exhibited relatively low average ages, with an average age at death of 59 years.

### 3.2 Persistence and Continuity of Cancer in Romania

**Figure 5:**
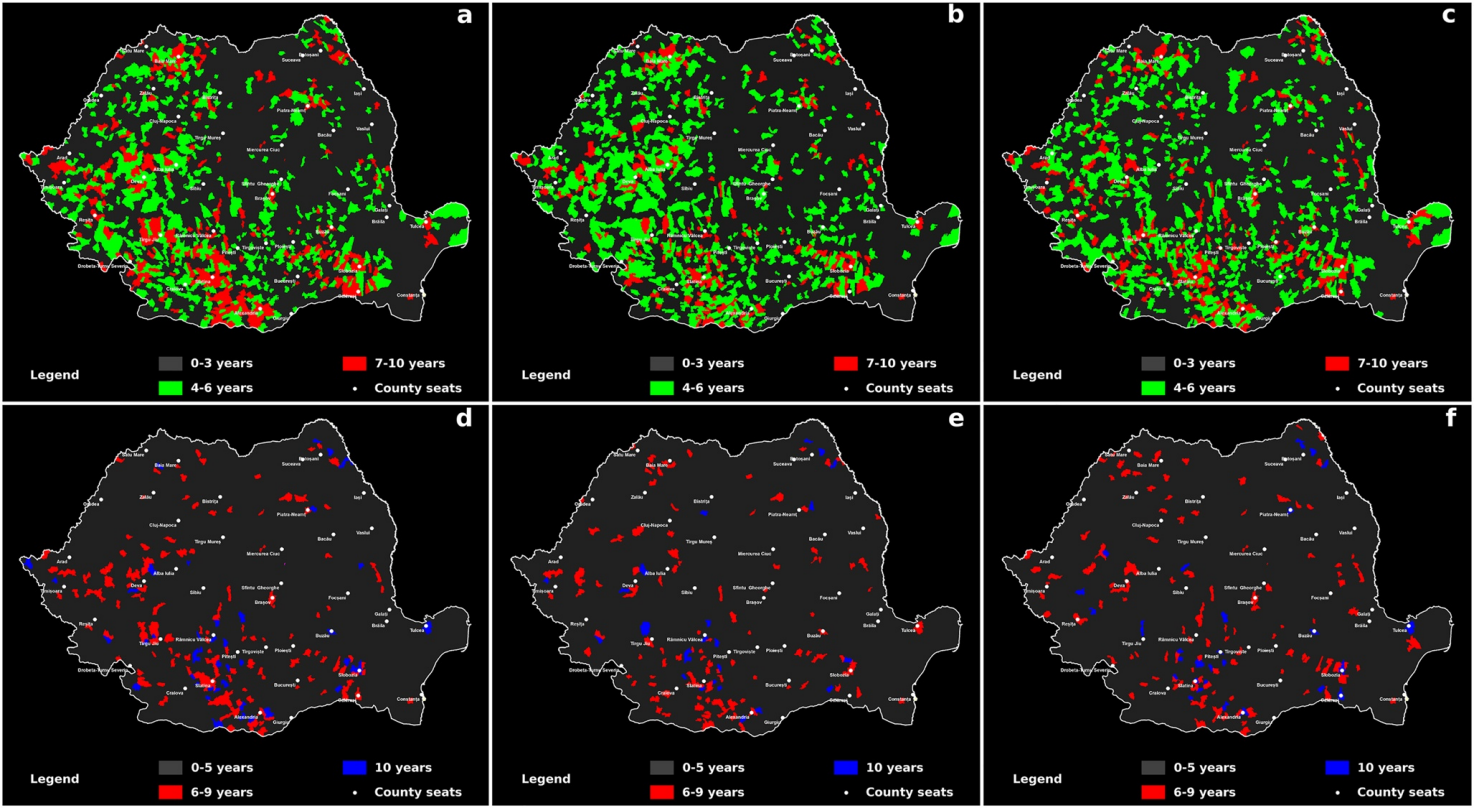
The persistence and continuity of cancer in Romania, focusing on various aspects. Panel (a) represents the persistence of cancer prevalence in the total population, while (b) and (c) illustrate the persistence of cancer in the male and female populations, respectively. Additionally, panels (d–f) showcase the continuity of cancer prevalence in the total population, male population, and female population, respectively.

Persistence is a parameter that highlights the duration of a phenomenon in a specific territory. Figure 5a–5c present the persistence of cancer prevalence at the level of TAU in Romania. Figure 5a displays the total cases in both genders, while Figure 5b focuses on the total numbers in the male population, and Figure 5c examines the female population.

Figure 5a reveals that out of the 3,181 TAU, 258 exhibited persistent cancer prevalence in the total population for 7–10 years, 677 units showed persistence for 4–6 years, and 2,246 units had persistence of less than 3 years. Among the developing regions, the South‐West Development Region had the highest persistence rate (24.3% of TAU), followed by the South Development Region (13.55%) and the West Development Region (13%). In contrast, the North‐East, Center, and South‐East regions displayed persistence rates below 5%. At the county level, the highest prevalence of cancer was observed in Olt, Gorj, Teleorman, Ialomița, Maramureș, Hunedoara, Mehedinți, Vâlcea, Botoșani, and Timiș counties, which accounted for over 15% of the TAU. Conversely, Brăila, Covasna, Mureș, Vaslui, Vrancea, and Bucharest had no recorded instances of maximum persistence in total cancer prevalence. These counties predominantly demonstrated a dominance of low persistence (0–3 years) in more than 84% of their TAU.

Figures 5b and 5c show similar trends, with a lower number of TAU and lower percentages compared to the total values. Figure 5b reveals that out of the 3,181 TAU, 229 exhibited persistent cancer prevalence in the male population for 7–10 years, 714 units showed persistence for 4–6 years, and 2238 units had persistence of less than 3 years. Notably, only a quarter of the TAU in the Bucharest‐Ilfov Development Region displayed high persistence of cancer prevalence in both total and male populations (1 out of 4 units). Moreover, it was evident that the persistence of cancer prevalence in the male population was lower than in the total population. The Center Development Region stood out with 16 TAU showing higher persistence compared to the total (14 units), indicating a dominance of male cancer persistence. Other developing regions had 13%–39% fewer TAU with persistent cancer prevalence in the male population. At the county level, the highest male cancer prevalence exceeding 15% of TAU occurred in Olt, Ialomița, Maramureș, Timiș, Călărași, Vâlcea, and Gorj counties. Conversely, Brăila, Giurgiu, Mureș, Vrancea, and Bucharest had no recorded instances of maximum persistence in male cancer prevalence. These counties primarily exhibited a dominance of TAU with low persistence (0–3 years) in over 87% of cases.

Figure 5c indicates that out of the 3,181 TAU, 258 displayed persistent cancer prevalence in the female population for 7–10 years, 677 units showed persistence for 4–6 years, and 2247 units had persistence of less than 3 years. Figure 5c highlights that only the South‐East Development Region had the highest number of TAU with maximum persistence of female cancer prevalence (18 units out of the total), compared to 8 units for male cancer and 13 units for the total population. Other development regions exhibited values between 8% and 39% less than the total number of TAU.

Comparing Figure 5a–5c, it is evident that the maximum persistence of female and male cancer incidents did not entirely coincide with the persistence of total cancer. This indicates that areas with persistent cancer prevalence in the female population might not have the same persistence in the male population, and vice versa. The North‐East Development Region was the only region where the maximum persistence occurred in 23 TAU for both female and male cancer. However, since these units did not coincide, there were 27 units with maximum persistence.

At the county level, the TAU with the highest cancer prevalence in the female population (exceeding 15% of the total units) were observed in Olt, Gorj, Ialomița, Teleorman, Călărași, and Maramureș counties. Conversely, the counties of Brăila, Bistrița‐Năsăud, Mureș, and Bucharest did not register maximum persistence of female cancer prevalence. These counties predominantly recorded a dominance of TAU with low persistence (0–3 years) in over 84% of cases.

The concept of continuity of persistence is introduced as a new analytical approach in this study. It highlights whether a phenomenon has persisted continuously for an extended period, constituting over 50% of the analyzed time. Figures 5d–5f illustrate the continuity of cancer prevalence in TAU in Romania for the total population, male population, and female population, respectively.

Figure 5d shows that out of the 3,181 TAU, 49 had continuous cancer prevalence for 10 years in the total population, 168 units exhibited prevalence over 6–9 years of persistence continuity, and 2,964 units had continuity of less than 5 years. Among the counties, Brăila, Covasna, Giurgiu, Iași, Mureș, Sibiu, Tulcea, Vaslui, Vrancea, and Bucharest had no TAU with a continuity of cancer prevalence lasting 6–9 years in the total population (0% occurrence). Only four counties (Olt, Teleorman, Hunedoara, and Gorj) had over 15% of TAU exhibiting continuity during 6–9 years of persistence.

Regarding the maximum continuity of 10 years, 23 out of 41/42 counties did not have a single TAU with complete continuity for the entire duration of analysis (10 years out of 10 years). Over 5% of the TAU exhibited continuity of persistence during 10 years in Olt, Vâlcea, Botoșani, and Teleorman counties.

At the level of development regions, the South‐West and West regions had the highest concentrations of TAU with continuity of persistence for 6–9 years, while the North‐East and South‐East regions had the lowest concentrations. For the continuity of persistence over 10 years, the South‐West and South regions had the highest numbers, while the North‐West and Bucharest‐Ilfov regions recorded 0% continuity.

Figure 5e reveals that out of the 3,181 TAU, 25 displayed continuity for 10 years of cancer prevalence in the male population, 124 units exhibited continuity for 6–9 years, and 3,032 units had continuity of less than 5 years. Only seven counties (Brăila, Giurgiu, Iași, Mureș, Sibiu, Vrancea, and Bucharest) had 0% TAU with a continuity of cancer prevalence lasting 6–9 years in the male population. Four counties (Olt, Maramureș, Teleorman, and Ialomița) had 9% of their TAU exhibiting continuity during 6–9 years of persistence. Concerning the maximum continuity of 10 years, 29 out of 42 counties did not record any TAU with complete continuity (10 years out of 10 years analyzed). Over 3% of the TAU exhibited continuity of persistence during 10 years in Vâlcea, Olt, and Botoșani counties. The South‐West, West, and South regions had the highest concentrations of TAU with continuity of persistence for 6–9 years, while the North‐East and South‐East regions had the lowest concentrations. In Bucharest‐Ilfov, Center, and South‐East regions, no TAU showed persistent continuity for 10 years.

Lastly, Figure 5f indicates that out of the 3,181 TAU, 30 units exhibited continuity of cancer prevalence for 10 years in the female population, 130 units showed continuity for 6–9 years, and 3,021 units had continuity of less than 5 years. Only four counties (Giurgiu, Iași, Mureș, and Bucharest) had 0% TAU with a continuity of cancer prevalence lasting 6–9 years in the female population. Four counties (Olt, Ialomița, Hunedoara, and Teleorman) had over 10% of their TAU exhibiting continuity during 6–9 years of persistence. Concerning the maximum continuity of 10 years, 27 out of 42 counties did not record any TAU with complete continuity (10 years out of 10 years analyzed). Over 3% of the TAU exhibited continuity of persistence during 10 years in Olt, Botoșani, and Călărași counties. The South‐West and West regions had the highest concentrations of TAU with continuity of persistence for 6–9 years, while Bucharest‐Ilfov, North‐East, and South‐East regions had the lowest concentrations. In the continuity of persistence for 10 years, the highest concentrations were noted in the South‐West and South regions. In the North‐West and Bucharest‐Ilfov regions, no TAU exhibited ongoing continuity for 10 years.

Overall, this study provides valuable insights into the persistence and continuity of cancer prevalence in Romania, shedding light on variations between gender, geographic regions, and timeframes. The findings highlight areas where cancer prevalence persists, offering potential areas for targeted interventions and healthcare planning.

## 4 Discussion

This study presents the first spatial geographical mapping of cancer fatality in Romania. The absence of a central database of geographical cancer distribution had previously hindered any attempts to conduct such a study. As a result, Romania was among the few countries in the European Union that had not benefited from such an analysis. The geographical perspective on the distribution of tumors in Romania offers a new direction of analysis that can contribute to understanding the complex relationships between environmental and socio‐economic conditions and tumor prevalence. The results obtained revealed high geographical concentrations of values, which requires further interdisciplinary research to synthesize the relational pattern between tumor occurrence and the specificities of the territorial reality. This spatial approach significantly contributed to our knowledge of the patterns of these relationships (Caudeville & Masson, 2008; Cicolella et al., 2008), and highlights two main lines of research: the relationship between environmental conditions and the continuity and persistence of high cancer values in certain geographical areas, and the relationship between the same parameters and industrial activities that have produced major imbalances in the environment.

The highest concentrations of malignant tumors were observed in major urban centers and large metallurgical industrial hubs known for their pollution during full operation. Due to the absence of ecological rehabilitation policies, these areas are still considered historically polluted and continue to pose environmental risks (Blain et al., 2013; Boscoe & Schymura, 2006; Ghetian et al., 2008; Mandal et al., 2009).

The spatial design of oncological prevalence and fatality complements standard oncological methods by incorporating new elements related to the natural and anthropogenic environment. Furthermore, this research was based on the patients' residential locations, providing a relevant depiction of carcinogenic geographical areas (Brewer, 2006; Crew & Neugut, 2006; Mosavi‐Jarrahi et al., 2007). The results obtained here pave the way for future investigations into geographical areas with low cancer rates. Additionally, this approach is expected to offer valuable insights into identifying environmental contributors to cancer. Subsequent research should focus on analyzing the geographical aspect of oncological prevalence and fatality for each type and subtype of tumor, aiming to identify new environmental factors associated with cancer development through detailed spatial analysis at the level of TAU. Furthermore, the use of nano structural approaches should be complemented with spatial autocorrelation projections, which is one of the most effective methods for analyzing the spatial patterns of natural and socio‐economic cancerogenic factors as it accounts for the complexity of both determinants and effects.

The primary contribution of this study lies in emphasizing the importance of researching the spatial dimension of oncological prevalence and fatality for evidence‐based health policy development and health economic analysis, particularly regarding early diagnosis and treatment. Moreover, the methodology employed in this research could contribute to the development of processes influenced by spatial factors (Andronache et al., 2017, 2019; Ciobotaru et al., 2019; Diaconu et al., 2019; Petrişor et al., 2016; Simion et al., 2021), as well as diagnostic methodologies that incorporate interdisciplinary approaches (Branisteanu et al., 2022; Busnatu et al., 2022; Halip et al., 2022; Ion et al., 2021; Ionescu et al., 2021; Paduraru et al., 2018).

The identification of geographical hotspots in cancer cases could decisively contribute to a better understanding of carcinogenesis by elucidating the role of environmental exposures, investigating cluster phenomena, exploring gene‐environment interactions, considering socioeconomic factors, facilitating data‐driven research and guiding the development of targeted prevention strategies (Sahar et al., 2019; Yomralioglu et al., 2009):

‐ A better understanding of carcinogenesis can be achieved by correlating the geographical distribution of potential environmental factors, such as air pollution, water contamination, radiation, or occupational hazards with cancer prevalence (Cani et al., 2023; Parsa, 2012).
‐ Clustering of cancer cases, revealed by analysis of geographical cancer hotspots, suggests localized causes or shared risk factors within areas of Romania. By investigating these clusters, researchers can explore commonalities in environmental, occupational, or lifestyle factors within the affected population. This investigation can help identify potential carcinogens or risk factors specific to certain geographical areas as identified in the current research (Amin et al., 2019; Goodman et al., 2014; Leiser et al., 2020).
‐ Geographical hotspots may coincide with regions where specific genetic mutations or variations are more prevalent. By examining the genetic profiles of individuals within cancer hotspots, researchers can explore the interplay between genetic factors and environmental exposures (Schierenbeck, 2017; Vintsek et al., 2022). This understanding of gene‐environment interactions can provide insights into the mechanisms through which genetic predisposition and environmental factors contribute to carcinogenesis (Hutter et al., 2013; Mbemi et al., 2020; Virolainen et al., 2022).
‐ In addition, examining hotspots with socioeconomic disparities, underlying mechanisms by which socioeconomic factors contribute to cancer development may be identified (Carethers & Doubeni, 2020; Ingleby et al., 2022; Lee et al., 2012; Redondo‐Sanchez et al., 2022).
‐ Geographical hotspot analysis also enables data‐driven research on carcinogenesis. By analyzing cancer cases within hotspots and comparing them to regions with lower prevalence, patterns, trends, and potential risk factors associated with cancer development can be identified that can drive targeted intervention (Dominguez et al., 2019). This data‐driven approach can generate hypotheses, guide further laboratory and epidemiological research, and facilitate the discovery of novel mechanisms and pathways involved in carcinogenesis (Kosvyra et al., 2020).
‐ Understanding the specific risk factors associated with geographical hotspots will allow policymakers to implement interventions aimed at reducing exposure to carcinogens, promoting healthy lifestyles, and improving access to preventive measures such as screening and vaccinations (Lofters et al., 2013; Wand & Ramjee, 2010).

In summary, focusing on prevention efforts in geographical hotspots could enable a better mitigation of carcinogenesis risks and reduce cancer incidence rates.

The main limitations of the research are the difficulties in updating data at the level of TAU, as well as the lack of relevant information on the number of cases at the level of some economic sectors, where it is suspected that they play an important role in increasing the number of cases. A major limitation is the mobility of the population which makes the link between tumor occurrence and environmental conditions difficult to analyze.

## 5 Conclusions

The obtained results show a decrease in the total number of cases with the C00–C96 categories but an increase in total mortality including an increase in female and male malignancies.

Primary malignant tumors located in the digestive organs (C15–C26) and respiratory and intrathoracic organs (C30–C39) accounted for the largest share of these cases. Furthermore, the detailed analysis of oncological mortality revealed an increase in the average age of individuals succumbing to cancer, particularly in the female population.

The investigation of persistence and continuity in cancer prevalence within the geographical space is crucial for understanding the relationship between environmental conditions and cancer occurrence. By setting the 75th percentile threshold interval, we were able to identify the underlying association between cancer and environmental factors. The pronounced disparities in the spatial distribution of cancer cases underscore the necessity for interdisciplinary research focused on identifying geographical and environmental contributors to carcinogenesis. The results obtained in this study lead to future research on the geographical areas with the lowest tumor burden, and the results may help validate the findings of this study.

This research emphasizes the significant influence of geographical factors on cancer prevalence. The geographical distribution of cancer cases plays a pivotal role in shaping national and European health policies related to cancer prevention, early detection, treatment, and resource allocation. By comprehending the variations in cancer incidence across different regions, policymakers can develop targeted strategies to alleviate the burden of cancer and enhance outcomes for affected individuals. Moreover, this understanding can contribute to a better grasp of the genetic, socioeconomic, and environmental factors contributing to cancer.

## Conflict of Interest

The authors declare no conflicts of interest relevant to this study.

## Data Availability

The data was obtained from the National School of Public Health, Management and Professional Development in Health (http://www.snspms.ro/). The data are public and are obtained following a written request to contact@snspms.ro. Data processing was conducted using the open‐source platform QuantumGIS (www.qgis.org).
